# The emerging role of cancer cell plasticity and cell-cycle quiescence in immune escape

**DOI:** 10.1038/s41419-020-2669-8

**Published:** 2020-06-18

**Authors:** Sara Bruschini, Gennaro Ciliberto, Rita Mancini

**Affiliations:** 1 Department of Experimental and Clinical Medicine, Magna Graecia University of Catanzaro, 88100 Catanzaro, Italy; 2 Scientific Directorate, IRCSS Regina Elena National Cancer Institute, 00128 Rome, Italy; 3 Department of Clinical and Molecular Medicine, Sapienza University of Rome, 00161 Rome, Italy

**Keywords:** Tumour immunology, Cancer

Molecular programs that control the function and the phenotype of stem cells are also active in cancer and confer properties that promote progression and resistance to therapy. Likewise, the specific properties that enable long-lived stem cells to evade immune surveillance can be co-opted by latent cancer cells responsible for tumour initiation as well as metastatic outbreak. Following the clinical success of anticancer immunotherapy in recent years, greater focus has been placed on the interplay between cancer cells and the tumour immune microenvironment. However, the link between stem-cell-like tumour phenotype and the immunological properties of cancer has not yet been systematically explored. New evidence discussed in this article, stemming from sophisticated approaches that make use of lineage tracing and single-cell analysis, provide strong proof of a link between cancer stem cells, tumour cell plasticity, cell-cycle quiescence and immune suppression in cancer^1^.

The concept that the immune system may have a protective role in tumour development found its roots in the immunosurveillance hypothesis by Burnet and Thomas^2,3^. New experimental data led to incorporating this hypothesis into the broader immuno-editing concept that consists in three phases: elimination, equilibrium and escape^4^. Along this view, the immune system exerts both host-protecting and tumour-sculpting effects and the pressure exerted by cancer immunosurveillance’s arsenal (i.e. elimination phase), together with the genetic instability of tumours, lead to an equilibrium phase. During this phase the best-fitting cancer cells, subjected to Darwinian selection, can lead to escape and the appearance of a clinically detectable disease^4^.

In the last few years, we have witnessed remarkable progress in the field of cancer immunology and today many of the mechanisms that tumours use to prevent clearance by the immune system have been revealed. However, the earliest events of immune evasion are not yet known. A set of recent studies showed that cancer stem cells (CSCs) have the ability to hide from the immune system ab initio, evading form the immunosurveillance phase. Agudo et al.^5^ showed that the immune privilege of epithelial stem cells is associated with their proliferative state and is not an inherent property they stably possess. To determine the interactions of T cells with adult tissue stem cells, in their niche, the authors injected in the Lgr5-GFP mice, Jedi (just EGFP death-inducing) T cells, which express a TCR specific for an immunodominant epitope of green fluorescent protein (EGFP), or control T cells as well as GFP (to activate Jedi T cells). Through using this clever approach, the authors showed that fast cycling stem cells, such as Lgr5+ stem cells in the gut, ovaries and mammary gland were subjected to immune clearance. In contrast, slow-cycling stem cells, such as those in hair follicles and muscle, were resistant to Jedi T-cell killing. Furthermore, immune escape depends on an intrinsic property of quiescent stem cells, which undergo a cell autonomous downregulation of the antigen presentation machinery mediated by the transactivator NLRC5. Notably, the process is reversible when stem cells enter the cell cycle. In addition, in ex vivo experiments, quiescent stem cells appear to be protected also from NK cell killing.

This ability of long-lived stem cells to evade immune surveillance is probably due to their critical role in the maintenance of tissue homeostasis. As CSCs can derive from normal stem cells, these findings suggest that CSCs may be the earliest cancer cells that evade immune surveillance, co-opting properties of quiescent stem cells. In this regard, Malladi et al.^6^ showed that the latency and immune evasion of metastatic cancer cells is strictly correlated with the acquisition of a slow-cycling stem-cell-like state. The researchers created a new metastatic model through injecting, in athymic mice, GFP-labelled lung and breast cancer cells and the subsequent isolation, after 3 months, in different organs of the mice of what they called latency competent cancer cells (LCC). By gene-set-enrichment analysis, the authors revealed that these cells show stem-like phenotype, express SOX2 and SOX9 transcription factors, and self-impose a slow-cycling state by the autocrine production of a WNT inhibitor, DDK1. Upon entering quiescence LCC cells evade NK-mediated immune surveillance through a broad downregulation of ULBP NK cells activators. In the proposed model, after infiltrating target organs, LCC proliferating cells are killed by NK cells, but a minority of them enter a quiescence state and remain latent for an extended time. As such, they retain a metastasis-initiating power as shown by NK depletion, a condition that may mimic a transient decrease in immune surveillance.

**Fig. 1:**
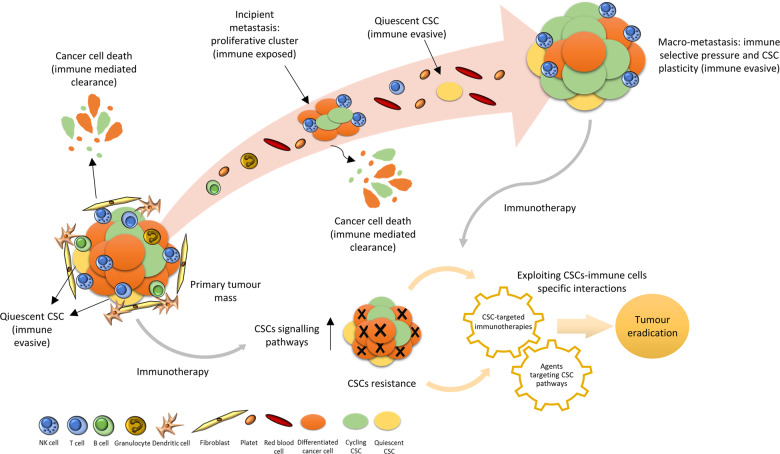
Quiescent cancer stem cells (CSCs) are able to evade immune surveillance and to promote tumour development. At the site of the primary tumour, differentiated cancer cells and proliferating clusters of CSCs are subjected to immune cells clearance, instead CSCs that enter in a quiescent state are hidden. Isolated quiescent CSCs can enter the bloodstream and, evading the surrounding immune cells, are able to exit from dormancy and colonize the metastatic site. Metastatic outbreak can occur thanks to CSC plasticity and the acquisition of new immune evasive mechanisms. Like other types of treatments, CSCs are also refractory to immunotherapies leading to tumour relapse. These resistant cells overexpress key antigens or metabolic vulnerabilities that can be targeted by newer CSCs-specific immunotherapies or drugs directed to CSCs-associated pathways. Immunotherapies could be combined with targeted CSCs agents to both debulk the original tumour and eradicate any emerging resistant cells. However, the optimal timing, sequence and combination of these CSCs-specific, immune-based therapies requires further studies.

Hence, the immune privileged status is not an intrinsic property of CSCs, but is linked to the ability to enter a quiescent condition. This was best demonstrated in a very recent study by the Massague’s lab^1^ in which through the use of single-cell RNA sequencing it was possible to identify in metastatic lesion of lung adenocarcinomas, cell subpopulations with different stem grading, depending on SOX2 and SOX9 expression ranging from adult stem cells state, intermediate regenerative subpopulations right through to alveolar proliferative progenitors. Taking advantage of the previously described LCC mouse model the authors demonstrated a developmental-stage-specific sensitivity to NK cells during the metastatic outbreak, suggesting CSC plasticity, that governs the ability to switch from one development program to another, as an alternative weapon to escape from NK cells and to survive (Fig. 1).

As CSCs are considered a subpopulation of tumour cells with enhanced capacity of self-renewal, metastatic dissemination and resistance to conventional treatment^7,8^, the studies discussed above explain in part how they may achieve these features. However, they do not specifically address issues related to resistance to immunotherapy. In this respect, Miao et al.^9^ showed that, in small-cell carcinoma (SCC), the TGF-β-responding CSCs, known to survive chemotherapy, are also refractory to adoptive T-cell transfer (ACT)-based-immunotherapy and are responsible for tumour relapse. Establishing a novel mouse SCC model in which neoantigen expression is coupled with oncogene activation and using lineage tracing, they identified a novel resistance mechanism to ACT therapy, that is not due to neoenatigen editing. In essence, employing single-cell RNA-Seq and in vitro co-culture analyses, the authors revealed that these CSCs selectively express the CD80 surface ligand that, upon engaging CTLA4 on T cells, mediates the exhaustion of their cytotoxic activity, after ACT treatment. The phenomenon is reversed upon CTLA4 or TGF-β-blocking immunotherapies, paving the way for a combination approach.

From the perspective that CSCs are not only the earliest but also the most resilient immune evaders, there is compelling urgency to identify approaches that selectively target this subpopulation of tumour cells. The main challenge in the CSCs research remains their isolation. Since the first postulation of their existence^10^, major efforts have been directed towards identifying specific surface as well as metabolic markers to discriminate between bulk tumour cells and CSCs. An example is represented by the increased lipid desaturation, sustained by SCD1 enzyme, in the stem-cell pool that has been proven in different tumour types^11,12^. In this framework, the specific interactions between CSCs and immune environment could also be exploited for detection and study of CSCs, a new approach adopted by Paczulla et al.^13^. These authors have identified AML stem cells by screening patients with acute myeloid leukaemia (AML) for expressing NKG2D ligands, known to be able to activate NK cells. The subset of NKG2DL-negative cells displayed molecular and functional properties of stem cells, and the ability to repopulate immunodeficient mice as well as to survive to chemotherapy in PDX models. Mechanistically in these cells high expression of PARP1 is responsible for the repression of NKG2DL, which mediates evasion from NK cells clearance. Moreover, pharmacologic inhibition of PARP1 resensitizes AML cells to NK cell killing in immunocompromised mice injected with human NK cells.

These findings, as well as those previously discussed, highlight the critical interplay between key signalling pathways crucial for stem-cells propagation and the mechanisms that guide immune evasion. Clinically this set of evidences open the intriguing perspective that therapeutic agents capable of targeting signalling pathways responsible for cell entry into quiescence could be combined with immunotherapies, based on the ability to harness the power of immune system to fight cancer cells^14^ (Fig. 1). In conclusion, a deeper understanding of the unique interactions between cancer stem cells and the immune system and the development of more sophisticated experimental systems could provide ground for the establishment of therapeutic strategies aiming to harness the immune system against the “hardest immune-evaders”.
